# Advantages of Using Blend Cultures of Native *L. plantarum* and *O. oeni* Strains to Induce Malolactic Fermentation of Patagonian Malbec Wine

**DOI:** 10.3389/fmicb.2018.02109

**Published:** 2018-09-06

**Authors:** Natalia S. Brizuela, Bárbara M. Bravo-Ferrada, Yolanda Curilén, Lucrecia Delfederico, Adriana Caballero, Liliana Semorile, M. Ángeles Pozo-Bayón, E. Elizabeth Tymczyszyn

**Affiliations:** ^1^Laboratorio de Microbiología Molecular, Instituto de Microbiología Básica y Aplicada, Departamento de Ciencia y Tecnología, Universidad Nacional de Quilmes, Bernal, Argentina; ^2^Facultad de Ciencia y Tecnología de los Alimentos, Universidad Nacional del Comahue y PROBIEN, CONICET-Universidad Nacional del Comahue, Neuquén, Argentina; ^3^Instituto de Investigación en Ciencias de la Alimentación, Consejo Superior de Investigaciones Científicas-Universidad Autónoma de Madrid, Madrid, Spain

**Keywords:** *L. plantarum*, *O. oeni*, Patagonian Malbec wine, flavor, L-malic acid

## Abstract

The malolactic fermentation (MLF) of Patagonian Malbec wine inoculated with blend cultures of selected native strains of *Lactobacillus plantarum* and *Oenococcus oeni* was monitored during 14 days, analyzing the strains ability to modify the content of some organic acids and to change the volatile compounds profile. The performance of the LAB strains was tested as single and blends cultures of both species. An implantation control by RAPD PCR was also carried out to differentiate among indigenous and inoculated strains. The *L. plantarum* strains UNQLp11 and UNQLp155 and the *O. oeni* strain UNQOe73.2 were able to remain viable during the monitoring time of MLF, whereas the *O. oeni* strain UNQOe31b showed a decrease of five log CFU at day 14. The four strains assayed showed a similar behavior in wine whether they were inoculated individually or as blend cultures. All strains were able to consume L-malic acid, particularly the *L. plantarum* strains, which showed the highest consumption values at day 14, both as single or blend cultures. The changes in the volatile compounds profile of Malbec wine samples, before and after MLF, were determined by HS-SPME and GC-MS technique. Wines inoculated with blend cultures containing strain UNQLp155 showed a decrease in the total alcohols content and an increase in the total esters content. On the other hand, wines inoculated with single cultures of strains UNQLp155, UNQOe31b or UNQOe73.2 showed no significant decrease in the total alcohols concentration but a significant increase in the total esters content. When strain UNQLp11 was inoculated as single or as blend culture with strain UNQOe31b, wines exhibited an increase in the total alcohols content, and a decrease in the total esters content. The content of diethyl succinate showed the greatest increase at final of MLF, and a particular synergistic effect in its synthesis was observed with a blend culture of strains UNQLp155 and UNQOe73.2. These results suggest that the use of blend cultures formulated with strains belonging to *L. plantarum* and *O. oeni* species could offer an interesting advantage to induce MLF in Malbec wines, contributing to diversify their aromatic profiles.

## Introduction

Lactic acid bacteria perform MLF, an important step in the red grapes winemaking. Wine deacidification is the main consequence of the conversion of L-malic to L-lactic acid, resulting in a decrease in titrable acidity of wine and a small increase of pH. MLF also leads to enhanced microbial stability and is usually believed to improve the complexity of the wine aroma (Pozo-Bayón et al., 2005; Renouf et al., 2005). The biosynthesis of aroma compounds during MLF includes the activity of a broad range of enzymes present in LAB, such as glycosidases, esterases, phenolic acid decarboxylases and citrate lyases, whose activities may affect wine aroma and complexity. Different studies have focused on the biosynthesis of aroma compounds during MLF and the concomitant organoleptic consequences (Ugliano et al., 2003; Costantini et al., 2009). On the other hand, the influence of LAB strains on wine aroma composition and complexity is not yet well-known. Different authors have shown that aroma/flavor wine attributes can vary according to LAB strains used in MFL induction (Gámbaro et al., 2001; Boido et al., 2009; Iorizzo et al., 2016; Cappello et al., 2017).

Although MLF often occurs spontaneously, by action of native LAB from grapes and cellar, it implies risks such as a considerable increase in the volatile acidity, consumption of residual sugars, and formation of undesirable metabolites such as biogenic amines (Mira de Orduña et al., 2000; Liu, 2002; Marcobal et al., 2006). In order to avoid losses in production, the use of commercial MLF starter cultures is normally recommended. However, the available commercial starters may have been formulated with LAB strains isolated from regions different to those in which they are going to be used and thus, adding a variability factor in wine production. The use of indigenous starter cultures best adapted to a specific wine-producing area is therefore recommended to maintain the wine regional characteristics (du Toit et al., 2011; Garofalo et al., 2015; Berbegal et al., 2016).

Malolactic fermentation of Patagonian red wines occurs spontaneously and the prevalence of strains belonging to *Oenococcus oeni* and *Lactobacillus plantarum* species during this winemaking step suggests that these strains are involved in leading the spontaneous MLF of Pinot noir and Merlot wines (Valdés La Hens et al., 2015). In previous works, a great number of strains of both species have been isolated and characterized regarding their oenological and technological properties (Bravo-Ferrada et al., 2013, 2014; Brizuela et al., 2017). A recent study regarding the behavior of selected native strains inoculated in sterile Pinot noir wine, and their ability to change the volatile compounds profile of wine (Brizuela et al., 2018), showed a decrease in the alcohols content and an increase in the volatile esters content, particularly when *O. oeni* strains were inoculated. Meanwhile, the *L. plantarum* strains were more efficient to consume the L-malic acid. With this background, the goal of this work was to study the effect of single or blend cultures of native strains of *O. oeni* y *L. plantarum* species to induce MLF of a Patagonian Malbec wine, investigating changes in the volatile compounds and in some organic acids content.

## Materials and Methods

### Wine Sample

A Patagonian Malbec wine vintage 2016, at final stage of AF (12.4% v/v ethanol, pH 3.6, <2.00 g/L residual sugars, 2.0 g/L-malic acid, 96 mg/L total SO_2,_ total acidity 3.98 g/L) was employed. In this wine, the AF was carried out with the native Patagonian F8 *Saccharomyces cerevisiae* strain (Simes et al., 2016).

### *O. oeni* and *L. plantarum* Strains

The selected Patagonian LAB strains used were: *L. plantarum* UNQLp11, and UNQLp155, and *O. oeni* UNQOe31b, and UNQOe73.2. These strains were isolated from Patagonian Pinot noir wines (vintages 2008 and 2014) and selected according to their oenological properties (Bravo-Ferrada et al., 2013, 2016; Brizuela et al., 2017).

### Cell Cultures and Acclimation

*Lactobacillus plantarum* strains were grown in MRS (Biokar Diagnostic, Beauvais, France) (De Man et al., 1960), and *O. oeni* strains were grown in MLO medium (Maicas et al., 1999). Bacterial cell cultures in the early stationary phase (∼10^9^ CFU/mL) were collected by centrifugation at 5,000 RPM for 10 min and suspended in the same volume of an acclimation medium (50 g/L MRS, 40 g/L D(-) fructose, 20 g/L D (-) glucose, 4 g/L L-malate, 1 g/L Tween 80, and 0.1 mg/L pyridoxine, adjusted to pH 4.6) supplemented with 6% v/v ethanol (Bravo-Ferrada et al., 2014). Cultures were incubated during 48 h at 21°C according to Brizuela et al. (2017).

### Vinification Assays at Laboratory Scale

Acclimated cells were harvested by centrifugation and inoculated (∼5 × 10^7^ CFU/mL) in 80 mL of wine to induce MLF. LAB strains were inoculated as single cultures (UNQLp11, UNQLp155, UNQOe31b, and UNQOe73.2) or blend cultures (UNQLp11/UNQOe31b, UNQLp11/UNQOe73.2, UNQLp155/ UNQOe31b, and UNQLp155/UNQOe73.2), and wine samples were incubated at 21°C during 14 days. For blend cultures only a half concentration of each strain was inoculated, in order to obtain a final concentration of ∼5 × 10^7^ CFU/mL. Control sample was not-inoculated Malbec wine, incubated in the same conditions that inoculated wine samples. Values of cell survival were determined by plating on MRS (*Lactobacillus*) or MLO (*O. oeni*) agar plates added with 100 mg/L of cycloheximide (Sigma, United States) and 20 mg/mL of nystatin (Sigma-Aldrich, Argentina).

### Implantation Strain Ability

Implantation strain ability in non-sterile Malbec wine samples was performed by Random Amplified Polymorphic DNA (RAPD) method. From each sample, before and after MLF, ten colonies were randomly chosen from MLO and/or MRS plates and inoculated in MLO or MRS broth, respectively, to obtain DNA from each culture. All colonies were characterized as LAB by Gram-positive staining, negative catalase, and morphology was observed. DNA extraction was performed according to Bravo-Ferrada et al. (2011). DNA samples were quantified using a Nanodrop spectrophotometer (Thermo Scientific, 1000) and visualized on a 1.0% (w/v) agarose gel. *Oenococcus* and *Lactobacillus* isolates were typed by RAPD-PCR analysis using primer M13 (Stenlid et al., 1994). Amplification reactions were performed according to Delfederico et al. (2006), and PCR products were separated on a 2.0% (w/v) agarose electrophoresis gel using a 100 bp ladder PB-L (Productos Bio-Lógicos, UNQ). The evaluation of implantation ability was performed by comparing the RAPD profiles of each colony with profiles of the inoculated strains.

### Organic Acids

Concentrations of L- malic, tartaric, citric, and L-lactic acids were measured at day 0 and 14th using the Enology BioSystems kits, according to manufacturer instructions (L-malic acid, Tartaric acid, Citric Acid, and Lactic acid, BioSystems SA, Barcelona, Spain).

### Headspace Solid Phase Microextraction (HS-SPME)

Headspace solid phase microextraction was employed for volatile compounds sampling following the protocols previously described (Rodríguez-Bencomo et al., 2011), with modifications. Briefly, 8 mL of wine or hydroalcoholic solution containing the aroma compounds were placed in a 20 mL headspace vial with 40 μL of the three internal standards (3-octanol, methylnonanoate, and 3,4-dimethylphenol), and sealed with a TFE/silicone septum (Supelco, Bellefonte, PA, United States). Samples were left in a water bath at 40°C for 5 min before the extraction. The extraction was performed with the exposure of a Stable Flex 85 μm carboxen–polydimethylsiloxane, CAR–DVB-PDMS fiber (Supelco) to the headspace of the sample for 10 min at 40°C and under constant stirring (500 rpm). After the extraction, the fiber was removed from the sample vial and desorbed in the GC injector port in splitless mode for 80 min. Six levels of concentration of each aroma compound (2, 10, 100, 500, 1000, 5000 μg/L), covering the concentration ranges expected in wines, were tested in duplicate. Prior to use, the fiber was conditioned following the supplier’s recommendation.

### Gas Chromatography–Mass Spectrometry Analysis

An Agilent 7890A GC system (Agilent, Palo Alto, CA, United States), with a split/splitless injector and interfaced with an Agilent 5975N mass spectrometer was used for volatile compounds analysis. The injector was set at 250°C. The Agilent MSD Chem Station Software (D.01.02 16 version) was used to control the system. Volatile compounds were separated on a DB-Wax polar capillary column (60 m × 0.25 mm i.d. × 0.50 μm film thickness) from Agilent (J&W Scientific, Folsom, United States). Helium was the carrier gas at a flow rate of 1 mL/min. The oven temperature was programmed as follows: an initial temperature of 40°C which was maintained during 5 min, and then increased to 240°C (4°C/min) which was kept for 15 min. For the MS system, the temperatures of the transfer line, quadrupole and ionization source were 270, 150, and 230°C respectively; electron impact mass spectra were recorded at 70 Ev ionization voltages and the ionization current was 10A. The acquisitions were performed in Scan (from 35 to 450 amu) and SIM modes. Peak identification was carried out by analogy of mass spectra with those of the mass library (Wiley 6.0 and NIST 2.0), and with those from reference compounds analyzed in the same conditions that wine samples. Quantitative data were obtained by calculating the relative peak area (or TIC signal) in relation to that of the three internal standards used (3-octanol, methylnonanoate and 3,4-dimethylphenol), depending on the volatile compound. Calibration curves of each compound were performed using a hydroalcoholic solution (pH 3.6, 14% v/v ethanol) spiked with the commercial pure reference compounds at six levels of concentration (2, 10, 100, 500, 1000, 5000 μg/L) covering the concentration ranges expected in wine and tested in duplicate.

The aroma standard solutions for the calibration curve were prepared in HPLC grade absolute ethanol supplied by Merck. The 51 compounds used were: butyl acetate (123-86-4), ethyl hexanoate (123-66-0), ethyl decanoate (110-38-3) and vanillin (121-33-5) from Merck (Darmstadt, Germany); isobutyl acetate (110-19-0), ethyl butanoate (105-54-4), isopentyl acetate (123-92-2), hexyl acetate (142-92-7), 1-hexanol (111-27-3), *cis*-3-hexen-1-ol (928-96-1), ethyl octanoate (106-32-1), furfural (98-01-1), linalool (78-70-6), γ-butyrolactone (96-48-0), diethyl succinate(123-25-1), α-terpineol (98-55-5), β-damascenone (23726-91-2), 2-phenylethyl acetate (103-45-7), geraniol (106-24-1), guaiacol (90-05-01), whiskey lactone (39212-23-2), α-ionone (79-77-6) and eugenol (97-53-0) from Sigma–Aldrich; hexanoic acid (142-62-1), and decanoic acid (334-48-5) from Scharlau (Barcelona, Spain) and 4-ethyl guaiacol (2785-89-9) from Lancaster (Eastgate, White Lund, Morecambe, England); α-pinene, β-pinene, limonene, terpinen-4-ol, β-citronellol, nerol, 5-methylfurfural, furfuryl alcohol, benzyl alcohol, β-phenylethyl alcohol, decanoic acid, 2,3-butanodione, ethyl propanoate, 1-butanol, ethyl 2-methylbutirate, *trans*-3-hexen-1-ol, β-ionone, γ-nonalactone, ethyl cinnamate, 4-ethylphenol, 2-methoxy-4-vinylphenol, 2,6-dimethylphenol, methyl vanillate, ethyl vanillate, acetovanillone, ethyl dodecanoate from Sigma–Aldrich. These compounds were selected for their important role for wine aroma, being representative of the wine volatile profile. The aroma standards were purer than 98%. All the solutions were stored at 4°C.

### Reproducibility of the Results

Three vinification assays were carried out using single or blend cultures and all the experiments, for each sample, were done, at least, in duplicate. The statistical analyses were carried out using GraphPad Prism 5 software (GraphPad Software Inc., San Diego, CA, 2007). Data are presented as mean ± SD and compared by one-way ANOVA followed by a Tukey or Dunnett post-test for multiple comparisons, and if *p* < 0.05 the difference was considered statistically significant.

## Results

Based on previous studies of oenological and technological behavior of native *L. plantarum* and *O. oeni* strains from Patagonian Pinot noir wines, by inoculation in wine-like media and in sterile wine, four strains were selected: two *L. plantarum* (UNQLp11, UNQLp155), and two *O. oeni* (UNQOe31b and UNQOe73.2). These strains were inoculated as single or blend cultures in samples of a non-sterile Patagonian Malbec wine, at final stage of AF, with the aim to analyze their ability to perform the MLF in presence of wine natural microbiota. The viable cell counts in Malbec wine samples at final stage of AF were ∼1 × 10^5^ CFU/mL of *Lactobacillus* and ∼1 × 10^4^ CFU/mL of *O. oeni.*

**FIGURE 1:**
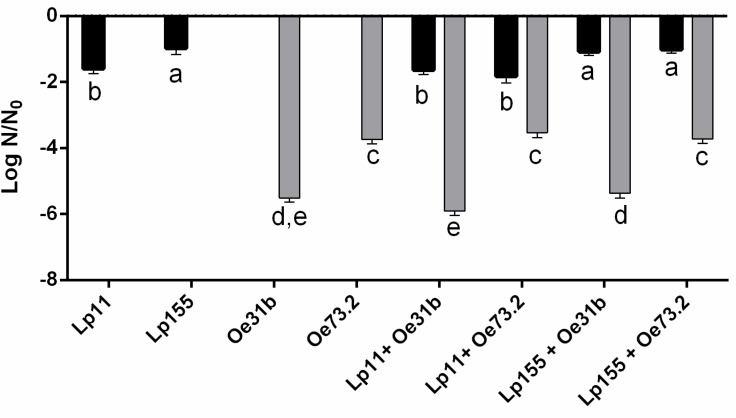
Loss of viability expressed as Log N/N_0_ of single cultures UNQLp11, UNQLp155, UNQOe31b, UNQOe73.2, and blend cultures UNQLp11/UNQOe31b, UNQLp11/UNQOe73.2, UNQLp155/UNQOe31b, and UNQLp155/UNQOe73.2 after 14 days of incubation in Malbec wine samples at 21°C. Gray bars indicate plate viable cell count number of strains belonging to *Oenococcus* species; black bars indicate viable cell counts of strains belonging to *Lactobacillus* species. Control wine showed no significant decrease in *Lactobacillus* count respect to day 0, but no *Oenococcus* species were detected after 14 days of MLF. Data are presented as mean ± SD and compared by one-way ANOVA followed by a Tukey post-test for multiple comparisons. Different letters (a–e) denote statistically significant difference (*p* < 0.05).

**Figure 1** shows the loss of cell viability of single and blend cultures (∼5 × 10^7^ CFU/mL) when were inoculated in Malbec wine and incubated during 14 days at 21°C. Values of viable cell counts of *O. oeni* strains showed a decrease of 4 to almost 6 log units, being the strain UNQOe31b less tolerant than strain UNQOe73.2. Instead, the viability of *L. plantarum* strains showed a lower decrease which did not exceed the 2 log units, being significantly lower for UNQLp155. For blend cultures the same behavior was observed as for single cultures. Blend cultures containing strain UNQOe31b exhibited the greatest loss of viability, whereas *L. plantarum* strains maintained a similar viability both as single and as blend cultures. In control wine sample (not inoculated), only *Lactobacillus* were detected after 14 days, indicating that *O. oeni* strains were not able to survive during this time. The implantation of cultures in non-sterile wine was controlled by RAPD PCR analysis with M13 primer. Percentages of implantation, at day 14, were: for single cultures, UNQLp11 25%, UNQLp155 43%, UNQOe31b 55%, and UNQOe73.2 23%, and for blend cultures, UNQOe31b (63%) + UNQLp11 (12.5%); UNQOe31b (60%) + UNQLp155 (25%); UNQOe73.2 (40%) + UNQLp11 (26%); UNQOe73.2 (37%) + UNQLp155 (35%) (data not shown).

**FIGURE 2:**
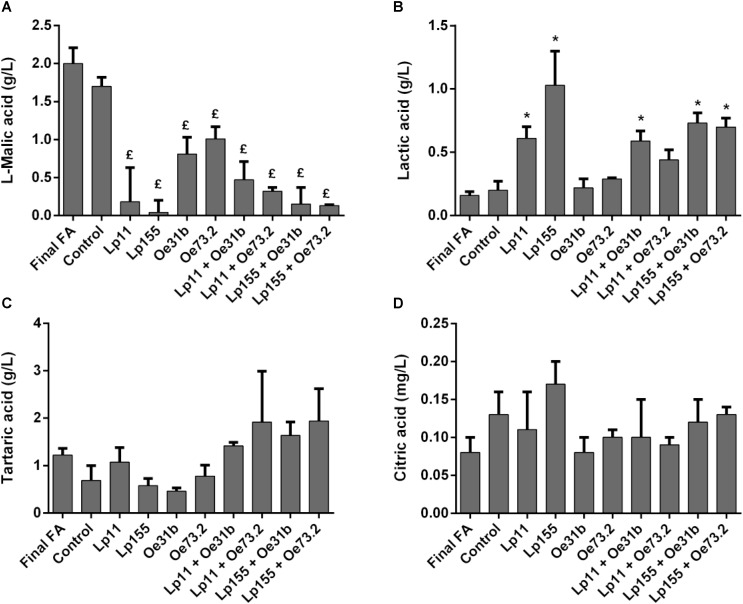
Remaining concentration of L-malic **(A)**, tartaric **(C)**, and citric **(D)** acids, and production of L-lactic acid **(B)**, measured after 14 days of wine samples incubation. Final FA refers to wine at day 0 of the assay. Control sample refers to non-inoculated wine at day 14. Data are presented as mean ± SD and compared by one-way ANOVA followed by a Dunnett post-test for multiple comparisons. (£) indicates significantly lower respect to control before MLF (*p* < 0.05); (^∗^) indicates significantly higher respect to control before MLF (*p* < 0.05). No significant differences in the tartaric and citric acid concentrations were observed (*p* > 0.05).

**Figure 2** shows changes in the concentrations of L- malic, L-lactic, tartaric, and citric acids, before and after, vinification assays. The four single cultures were able to significantly consume L-malic acid, being UNQLp155 and blend cultures containing this strain those who consume the largest amount (**Figure 2A**). Control wine sample showed no significant consumption of L- malic acid, indicating the failure or delay of natural microbiota to conduct MLF.

Regarding L-lactic acid production (**Figure 2B**), only single cultures of *L. plantarum* strains (UNQLp11 and UNQLp155) and blend cultures (UNQLp11/UNQOe31b, UNQLp155/UNQOe31b, and UNQLp155/UNQOe73.2) were able to significantly increase its concentration during vinification assays. While the single culture UNQOe73.2 and blend culture UNQLp11/UNQOe73.2 were able to consume 50 and 85%, respectively, of L-malic acid, the increase in L-lactic acid concentration was not significant. In relation to changes in the content of tartaric (**Figure 2C**) and citric acids (**Figure 2D**), although they seem to show remarkable variations, there were no significant for any of cultures assayed (*p* > 0.05).

**FIGURE 3:**
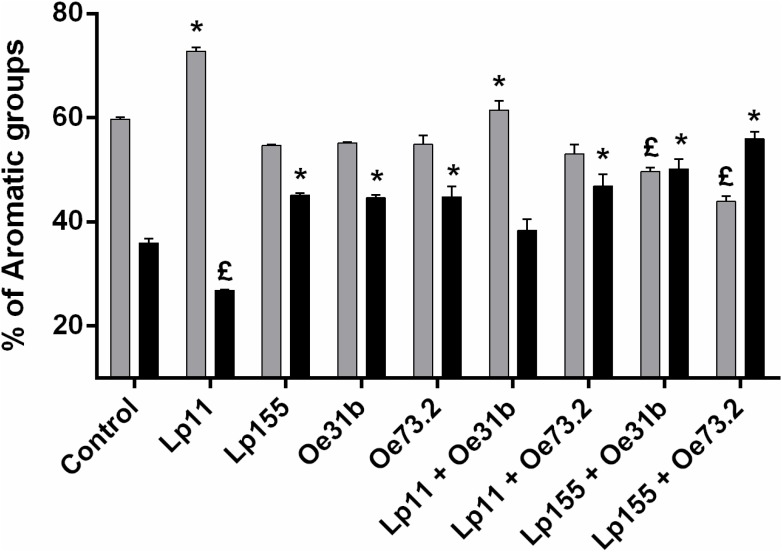
Changes in the percentage of total alcohols (gray bars) and total esters (black bars) concentrations in the Malbec wine samples inoculated wiLp155, UNQOe31b, and UNQOe73.2, and the blend cultures UNQLp11/UNQOe31b, UNQLp11/UNQOe73.2, UNQLp155/UNQOe31b, and UNQLp155/UNQOe73.2, or not inoculated (control). (£) indicates significantly lower respect to control before MLF; (^∗^) indicates significantly higher respect to control before MLF.

Modifications in the volatile compounds profiles of Malbec wine samples were determined by HS-SPME gas chromatography technique. Changes in concentration of 5 alcohols, 9 esters, 1 terpene, and 3 other volatile compounds, before and after vinification assay, were monitored (**Tables 1**, **2** and **Figure 3**). Alcohols were the main volatile compounds in Malbec wine samples at final stage of AF. Although a decrease of 3-methyl-1-butanol, 1-butanol, and 1-hexanol was observed with the different cultures inoculated, the percentage of total alcohols only decreased when MLF was carried out by the single culture UNQLp155 and blend cultures UNQLp155/UNQOe31b, and UNQLp155/UNQOe73.2, noting also an increase in total esters content. Wine samples inoculated with the other three single cultures (UNQLp11, UNQOe31b, and UNQOe73.2) showed no significant changes in total alcohols content, but a significant increase in total esters content was observed. Wine sample inoculated with blend culture UNQLp11/UNQOe31b exhibited an increase in total alcohols content, and the one inoculated with single culture UNQLp11, showed a significant decrease in total esters content (**Tables 1**, **2** and **Figure 3**).

**Table 1:** Volatile compounds content (mg/L) in wine samples after (day 14) inoculation with cultures of the single strains.

Aromatic compounds (mg/L)	Control wine	*L. plantarum*		*O. oeni*	
		UNQLp11	UNQLp155	UNQOe31b	UNQOe73.2
**Alcohols**					
3-methyl-1-butanol	1.56 ± 0.18	0.71 ± 0.02 (£)	0.48 ± 0.02 (£)	0.53 ± 0.01 (£)	0.59 ± 0.03 (£)
1-butanol	4.28 ± 0.10	1.58 ± 0.07 (£)	nd (£)	1.07 ± 0.02 (£)	1.14 ± 0.02 (£)
1-hexanol	3.78 ± 0.06	1.09 ± 0.02 (£)	0.81 ± 0.01 (£)	0.95 ± 0.05 (£)	1.09 ± 0.17 (£)
Benzyl alcohol	Nd	0.12 ± 0.14	0.12 ± 0.01	nd	nd
β-phenyl ethyl alcohol	9.84 ± 0.09	8.93 ± 0.45	7.74 ± 0.19	10.01 ± 0	9.93 ± 1.35
**Esters**					
Isobutyl acetate	0.44 ± 0.05	0.11 ± 0.01 (£)	0.08 ± 0.01 (£)	0.09 ± 0.01 (£)	0.10 ± 0.01 (£)
Ethyl butyrate	1.33 ± 0.07	0.37 ± 0.03 (£)	0.29 ± 0.01 (£)	0.31 ± 0.03 (£)	0.35 ± 0.07 (£)
Isoamyl acetate	3.59 ± 0.15	0.99 ± 0.04 (£)	0.78 ± 0.01 (£)	1.01 ± 0.24 (£)	1.24 ± 0.48 (£)
Ethyl hexanoate	1.97 ± 0.11	0.80 ± 0.02	0.72 ± 0.05 (£)	0.87 ± 0.09	1.04 ± 0.32
Hexyl acetate	0.03 ± 0.08	0.01 ± 0	0.02 ± 0.01	0.017 ± 0	0.023 ± 0
Ethyl octanoate	1.45 ± 0.05	0.76 ± 0.04	0.07 ± 0.01 (£)	0.82 ± 0.03	0.46 ± 0.46 (£)
Ethyl decanoate	1.79 ± 0.17	0.74 ± 0.01 (£)	0.06 ± 0.03 (£)	0.748 ± 0.06 (£)	0.43 ± 0.49 (£)
Diethyl succinate	0.73 ± 0.15	0.49 ± 0.02	5.46 ± 0.31 (^∗^)	5.95 ± 0.11 (^∗^)	6.44 ± 0.20 (^∗^)
Ethyl dodecanoate	0.25 ± 0.08	0.26 ± 0.01	0.04 ± 0.01 (£)	0.25 ± 0.01	0.24 ± 0
**Terpenes**					
β-citronellol	Nd	0.02 ± 0 (^∗^)	nd	nd	0.02 ± 0.02 (^∗^)
**Other compounds**					
2,3-butanodione	1.33 ± 0.17	nd (£)	nd (£)	nd (£)	nd (£)
γ-butyrolactone	9.99 ± 0.21	7.72 ± 0.81	7.31 ± 1.93	7.62 ± 1.44	8.20 ± 1.35
Furfural	0.09 ± 0.03	0.04 ± 0 (£)	0.03 ± 0 (£)	0.04 ± 0 (£)	0.04 ± 0 (£)

*(^∗^): Significantly higher respect to control before MLF (P< 0.05), (£): Significantly lower respect to control before MLF (P < 0.05). nd: not detected. Control wine correspond to non-inoculated wine.*

**Table 2:** Volatile compounds content (mg/L) in wine samples after (day 14) inoculation with cultures of blend cultures.

Aromatic compounds (mg/L)	Control wine	*Mixed cultures L. plantarum + O. oeni*			
		UNQLp11 + UNQOe 31b	UNQLp11 + UNQOe 73.2	UNQLp155 + UNQOe 31b	UNQLp155 + UNQOe 73.2
**Alcohols**					
3-methyl-1-butanol	1.56 ± 0.18	0.58 ± 0.11 (£)	0.65 ± 0.05 (£)	0.54 ± 0.07 (£)	0.77 ± 0.21 (£)
1-butanol	4.28 ± 0.06	1.22 ± 0.09 (£)	1.42 ± 0.15 (£)	1.145 ± 0.15 (£)	1.73 ± 0.40 (£)
1-hexanol	3.78 ± 0.10	1.12 ± 0.40 (£)	1.06 ± 0.07 (£)	0.98 ± 0.01 (£)	1.184 ± 0.15 (£)
Benzyl alcohol	Nd	0.22 ± 0.07	0.20 ± 0.02	0.13 ± 0.01	0.14 ± 0.04
β-phenyl ethyl alcohol	9.84 ± 0.09	8.88 ± 1.06	8.64 ± 1.61	9.11 ± 0.56	9.33 ± 0.27
**Esters**					
Isobutyl acetate	0.44 ± 0.05	0.13 ± 0 (£)	0.10 ± 0.01 (£)	0.08 ± 0 (£)	0.10 ± 0 (£)
Ethyl butyrate	1.33 ± 0.07	0.36 ± 0.15 (£)	0.32 ± 0.02 (£)	0.30 ± 0.02 (£)	0.37 ± 0.04 (£)
Isoamyl acetate	3.59 ± 0.15	0.90 ± 0.34 (£)	0.83 ± 0.08 (£)	0.73 ± 0.02 (£)	1.19 ± 0.39 (£)
Ethyl hexanoate	1.97 ± 0.11	0.98 ± 0.28	0.86 ± 0.22	0.93 ± 0.12	0.97 ± 0.32
Hexyl acetate	0.03 ± 0.08	0.02 ± 0	0.01 ± 0	0.01 ± 0	0.02 ± 0
Ethyl octanoate	1.45 ± 0.05	0.84 ± 0.28	0.83 ± 0.16	1.13 ± 0.15	0.77 ± 0.17
Ethyl decanoate	1.79 ± 0.17	0.93 ± 0.07 (£)	0.86 ± 0.01 (£)	0.96 ± 0.02 (£)	0.92 ± 0.28 (£)
Diethyl succinate	0.73 ± 0.15	2.98 ± 1.02	6.45 ± 1.75 (^∗^)	7.56 ± 1.56 (^∗^)	12.11 ± 1.29 (^∗^)
Ethyl dodecanoate	0.25 ± 0.08	0.26 ± 0.01	0.28 ± 0.02	0.29 ± 0.01	0.27 ± 0.02
**Other compounds**					
2,3-butanodione	1.33 ± 0.17	nd (£)	nd (£)	nd (£)	nd (£)
γ-butyrolactone	9.99 ± 0.21	nd (£)	6.91 ± 1.10	7.51 ± 0.50	7.55 ± 2.50
Furfural	0.09 ± 0.03	0.03 ± 0.005 (£)	0.04 ± 0 (£)	0.04 ± 0 (£)	0.04 ± 0 (£)

*(^∗^): Significantly higher respect to control before MLF (P < 0.05), (£): Significantly lower respect to control before MLF (P < 0.05). nd.: not detected. Control wine correspond to non-inoculated wine.*

As can be seen in **Table 1**, all volatile compounds tested showed a decrease in its concentration values when vinification assay was carried out by single cultures, while esters content (**Figure 3**) showed an upward trend (except with UNQLp11 culture) respect to control wine. It is important to note that diethyl succinate was the only volatile compound that showed an increase in its concentration at day 14th with most of inoculated cultures, except with UNQLp11 and the blend UNQLp11/UNQOe31b. The single cultures UNQLp155, UNQOe31b and UNQOe73.2 showed values of diethyl succinate concentration of 5.46, 5.95 and 6.44 mg/L, respectively, at day 14, while blend cultures UNQLp11/UNQOe73.2, UNQLp155/UNQOe31b, and UNQLp155/UNQOe73.2 showed higher values, being these 6.45, 7.56 and 12.11 mg/L, respectively. It should be noted the remarkable synergistic effect showed by blend culture UNQLp155 /UNQOe73.2.

On the other hand, all single or blend cultures were able to significantly reduce the furfural content in wine samples (**Tables 1**, **2**). In relation to the volatile compound β-citronellol, it was detected in wine samples inoculated with the single cultures UNQLp11 and UNQOe73.2, but not when blend cultures of these strains were inoculated (**Tables 1**, **2**). Finally, the presence of diacetyl (2,3-butanodione) could not be detected in any of the wine samples (**Tables 1**, **2**).

## Discussion

The results obtained in this work suggest that inoculation of a non-sterile Patagonian Malbec wine with single or blend cultures of selected native LAB strains, improves the performance of MLF in vinification assays at laboratory scale. Respect to consumption of L-malic acid, cultures containing *L. plantarum* strains (as single or blend cultures) showed greater ability than those containing only *O. oeni* strains, a result that agrees with a better survival in wine of *L. plantarum* strains. The implantation analysis showed the presence of the inoculated strains after 14 days of incubation, but percentages of implantation were lower than 100%, suggesting that inoculated strains did not have an inhibitory effect on the wine natural microbiota. In addition, although changes in organic acid and volatile compounds profiles could have been due to the microbial community of the wine sample (natural microbiota and inoculated strains), the highest consumption of L-malic acid was only observed when wine was inoculated with LAB cultures.

Although many authors have studied the control and improvement of MLF by inoculation of single LAB starter cultures (Maicas et al., 1999; Ugliano et al., 2003; Pozo-Bayón et al., 2005; Costello et al., 2012; Garofalo et al., 2015; Iorizzo et al., 2016) and mixed *O. oeni* strains starter cultures (Carreté et al., 2006), as to our knowledge, no previous works have analyzed the effects of inoculating blend cultures of *O. oeni* and *L. plantarum* strains. Only one starter culture containing strains of these two LAB species is provided at commercial level, but these strains come from a different wine region that Argentinean North-Patagonia, and the employment of autochthonous strains of a specific wine-producing area has been strongly recommended by several author, as we mention in the introduction section (Ruiz et al., 2010; du Toit et al., 2011; Garofalo et al., 2015; Berbegal et al., 2016).

Several authors have reported that MLF improves wine flavor by reducing herbaceous notes, due to C6 alcohols content, and enhances the fruity aroma, increasing esters content (Girard et al., 1997; Peinado et al., 2004; Costello et al., 2012; Feng et al., 2017). Inoculation of Malbec wine with autochthonous *L. plantarum* and *O. oeni* cultures, with the exception of those containing strain UNQLp11, showed an increase in esters content. While concentrations of some esters showed a decrease, such as ethyl butyrate, isoamyl acetate, ethyl hexanoate, ethyl octanoate and ethyl decanoate, all Malbec wine samples showed values above the sensory threshold, which has been related to some fruity aromatic notes (strawberry, banana and apple odor) (Peinado et al., 2004; Feng et al., 2017). Additionally, a notable increase in diethyl succinate concentration was observed when wine samples were inoculated with single cultures of strains UNQLp155, UNQOe31b and UNQOe73.2 (5.46, 5.95 and 6.44 mg/L, respectively) and with blend cultures. Particularly, a synergistic effect was detected between strains UNQOe73.2 and UNQLp155 were inoculated as blend culture. An increase in diethyl succinate concentration is related to fruity and melon odor (Peinado et al., 2004) and was previously reported as a positive characteristic in several types of wines (Goldner et al., 2011; Knoll et al., 2012). Also, it has been described that reduction of furfural wine content, by LAB inoculation, contributes to diminish the caramel-like odor notes (Hale et al., 1999).

Strains UNQLp11 and UNQOe73.2 were also able to produce β- citronellol, which is an odorant terpene released by β- glucosidase activity, previously described in some LAB (Maturano and Saguir, 2017). Although this compound was below the sensory threshold, both strains seemed to have β-glucosidase activity that allowed aroma precursors hydrolysis and release of odorant aglycones, such as terpenes alcohols with pleasant floral odor properties. The development of new starter cultures able to improve the aromatic qualities of wine is required and further studies regarding this activity are needed.

On the other hand, it was shown that strain UNQLp155 was able to survive in Malbec wine during 14 days of MLF, whereas the viability of this strain in Pinot noir wine was lower (Brizuela et al., 2018). The difference in cell survival could be due to ethanol content of both wines [which is lower in Malbec wine (12.4% v/v) than in Pinot noir wine (14.5% v/v)], since ethanol can induce disruption of membrane cell integrity, and consequently a higher mortality rate (Bravo-Ferrada et al., 2014).

## Conclusion

In conclusion, our results reveal a different malolactic behavior of single and blend LAB cultures. Regarding changes in the wine volatile compounds profile, *O. oeni* strains were able to produce higher amounts of significant volatile compounds due to their odor characteristics, indicating an active metabolism despite its lower viability compared to *L. plantarum* strains. The presence of *L. plantarum* strains in blend cultures guaranteed a higher consumption of L-malic acid, while *O. oeni* strains provide a greater capacity to change wine volatile compounds profile. The employ of such blend cultures to induce MLF in Malbec wines could offer an interesting advantage to improve the sensory attributes and quality of wine.

## Author Contributions

NB and BB-F did the experimental work regarding malolactic fermentation (culturability, organic acid concentrations, and implantation of LAB strains). NB and MP-B did the determination of volatile compound profile and analyzed results obtained by HS-SPME and GC-MS technique. YC, LD, and AC did the experimental work regarding alcoholic fermentation. LS and ET coordinated the work (analysis of results, discussion, and writing of the manuscript). All authors have approved the final version of the manuscript.

## Conflict of Interest Statement

The authors declare that the research was conducted in the absence of any commercial or financial relationships that could be construed as a potential conflict of interest.

## Abbreviations

AF: Alcoholic fermentation
GC-MS: gas chromatography-mass spectrometry
HS-SPME: Headspace solid phase microextraction
LAB: Lactic Acid Bacteria
MLF: Malolactic Fermentation.
